# Microbial Diversity in the Midguts of Field and Lab-Reared Populations of the European Corn Borer *Ostrinia nubilalis*

**DOI:** 10.1371/journal.pone.0021751

**Published:** 2011-06-30

**Authors:** Eugeni Belda, Laia Pedrola, Juli Peretó, Juan F. Martínez-Blanch, Arnau Montagud, Emilio Navarro, Javier Urchueguía, Daniel Ramón, Andrés Moya, Manuel Porcar

**Affiliations:** 1 Institut Cavanilles de Biodiversitat i Biologia Evolutiva, University of Valencia, Valencia, Spain; 2 Instituto Universitario de Matemática Pura y Aplicada, Universitat Politècnica de València, Valencia, Spain; 3 Lifesequencing, Valencia, Spain; 4 Departamento de Lenguajes y Ciencias de la Computación, Campus de Teatinos, Universidad de Málaga, Málaga, Spain; Université Paris Sud, France

## Abstract

**Background:**

Insects are associated with microorganisms that contribute to the digestion and processing of nutrients. The European Corn Borer (ECB) is a moth present world-wide, causing severe economical damage as a pest on corn and other crops. In the present work, we give a detailed view of the complexity of the microorganisms forming the ECB midgut microbiota with the objective of comparing the biodiversity of the midgut-associated microbiota and explore their potential as a source of genes and enzymes with biotechnological applications.

**Methodological/Principal Findings:**

A high-throughput sequencing approach has been used to identify bacterial species, genes and metabolic pathways, particularly those involved in plant-matter degradation, in two different ECB populations (field-collected vs. lab-reared population with artificial diet). Analysis of the resulting sequences revealed the massive presence of *Staphylococcus warneri* and *Weissella paramesenteroides* in the lab-reared sample. This enabled us to reconstruct both genomes almost completely. Despite the apparently low diversity, 208 different genera were detected in the sample, although most of them at very low frequency. By contrast, the natural population exhibited an even higher taxonomic diversity along with a wider array of cellulolytic enzyme families. However, in spite of the differences in relative abundance of major taxonomic groups, not only did both metagenomes share a similar functional profile but also a similar distribution of non-redundant genes in different functional categories.

**Conclusions/Significance:**

Our results reveal a highly diverse pool of bacterial species in both *O. nubilalis* populations, with major differences: The lab-reared sample is rich in gram-positive species (two of which have almost fully sequenced genomes) while the field sample harbors mainly gram-negative species and has a larger set of cellulolytic enzymes. We have found a clear relationship between the diet and the midgut microbiota, which reveals the selection pressure of food on the community of intestinal bacteria.

## Introduction

Insects and related arthropods are a source of molecules of biotechnological interest, from the high-performing silk of spiders and silkworms, which is mimicked in the so-called bioinspired materials [1], to cellulolytic enzymes for biofuel production or paper waste treatment. Additionally, insect-associated microorganisms, particularly endosymbionts, are known to produce bioactive compounds that protect the host against adverse environmental conditions, predators or competitors and they have thus been suggested as suitable for biotechnological applications [2]. Most insects are phytophagous, and they harbor a microbiota specialized in the hydrolysis and fermentation of plant biomass in their guts. An obvious biotechnological application of this ecosystem lies in the identification and characterization of target molecules involved in lignocellulosic degradation, by way of next-generation sequencing technologies [3]. Metagenomics is a powerful tool that can reveal the genomic diversity of natural environments but, up to date, only a few reports exist on metagenomics of insects' intestinal tracts. One of the few is the termite *Nasutitermes* spp., the metagenomic analysis of which revealed that the Spirochetes and Fibrobacteres, present in the termite hindgut, are responsible for lignocellulose degradation activities [4]. A recent report on the proteome of this species identified 866 proteins, 197 of which had identified enzymatic activity [5]. However, the activity associated with these enzymes was proposed to be a consequence of the symbiotic relationship between the hindgut microbial community and its termite host, rather than a reflection of its role as cellulose degradation machinery. Besides termites, other taxa such as Coleoptera have been subject to high-throughput metagenomics studies to characterize their midgut bacteria [6]. However, to the best of our knowledge, although metagenomic libraries from the Lepidoptera-associated microbiome have been screened [7], there are no complete metagenomic sequencing reports on Lepidoptera.

The intestinal tract of Lepidoptera is an atypical environment. In contrast with the acidic digestive tracts of other insects, Lepidopteran midguts are extremely alkaline, within pH range 10–11 [8]. Not surprisingly, the activity of the enzymes present in the midgut of Lepidoptera, such as α-amylases, has been found to be optimal at alkaline values [9]. Accordingly, it seems reasonable to suppose that the strongly alkaline lepidopteran midgut may play a critical role in the type of microbial community it harbors.

In the present work, high-throughput sequencing has been used to characterize the microbiome associated with a major Lepidopteran pest, the European Corn Borer (ECB), *Ostrinia nubilalis*. In order to define the relationship between diet and microbial diversity, we focused on two different populations: a natural one, feeding on crops (corn and pepper); and a laboratory-reared population fed on a starch-rich artificial diet over generations. Here we present the results of the first metagenomic characterization of a Lepidopterous pest, which may shed light on the poorly understood host-microbiome-environment interactions. Furthermore, these results provide a set of sequences corresponding to plant-fiber degrading enzymes, which may have industrial applications requiring optimum alkaline activities.

## Results

### Taxonomic composition of *O. nubilalis* metagenomes as deduced by sequence reads

The objective of this study was to characterize the gut microbiome of two different populations of the Lepidoptera *O. nubilalis*: one reared in the laboratory on an artificial diet and a wild population collected from the field. With this data, we aimed to evaluate the influence of the environmental conditions and nutritional sources on the composition of the midgut-associated microbiome. To do so, a metagenomic approach was carried out on both insect populations, which involved direct pyrosequencing of microbial DNA from the dissected guts of each insect population with a previous enrichment step on solid media. For the lab-reared population, a total of 437,501 reads were generated representing a total of 136.42 Mb of DNA sequence, with an average sequence length of 311.82 bp. For the field population, a total of 538,480 reads were obtained representing a total of 190.32 Mb, with an average sequence length of 353.45 bp.

**Figure 1:**
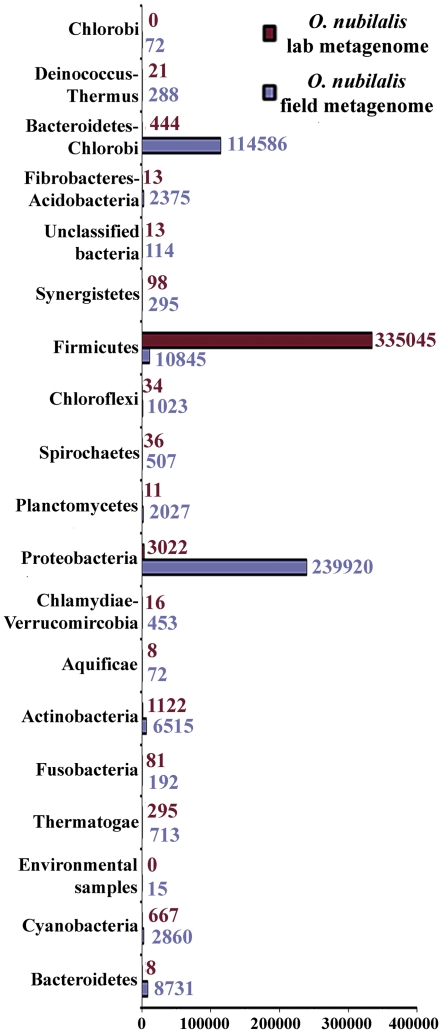
Taxonomic bining of *O. nubilalis* midgut metagenomes based on single reads. Total reads assigned to bacterial phyla in MG-rast analysis through BLASTX analysis against internal SEED protein database (maximum e-value cutoff 1×10−5).

**Figure 2:**
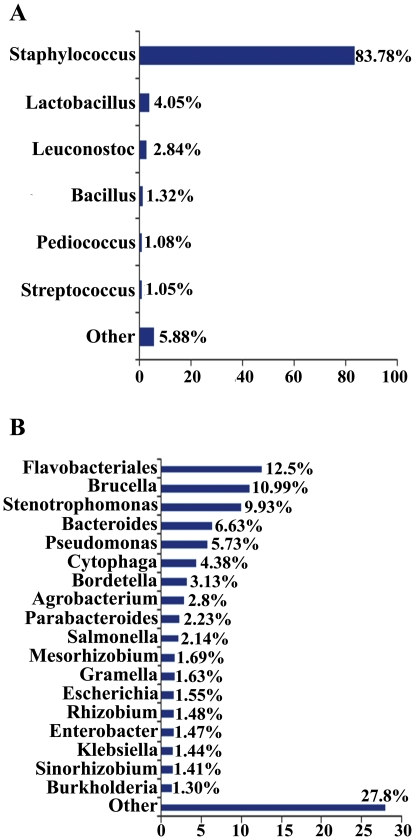
Comparison of taxonomic profiles of *O. nubilalis* midgut metagenomes at genera level. Fraction of reads assigned to different bacterial genera in MG-RAST analysis through BLASTX against internal SEED protein database (maximum e-value cutoff 10^−5^). (A) *O. nubilalis* lab metagenome; (B) *O. nubilalis* field metagenome.

An initial survey of the taxonomic profiles of both metagenomic datasets revealed the massive presence of reads assigned to bacteria in both metagenomes, although major differences in the relative abundance of the major phyla were observed (Figure 1). The BLASTX analysis of the sequence reads of the lab-reared population against SEED internal protein database matched 59% of the sequences at significant levels, 98.31% of which were bacterial while a minor fraction (0.64%) was of eukaryotic origin. Similar results were observed with sequence reads of the field population, where 59.3% proteins matched at a significant level (maximal e-value 1×E-5), 98.16% of which were of bacterial origin whereas 0.59% were assigned to eukaryotes and 0.95% were of viral origin. In the bacterial fraction of the lab-reared metagenome, sequences corresponding to the phylum Firmicutes predominated (98.54% of total bacterial reads) followed by sequences of the phylum Proteobacteria, although to a much lower extent (0.7%). In contrast, Proteobacteria proved to be the predominant phylum in the bacterial fraction of the field-population metagenome (62% of the bacterial reads) followed by the Bacteroidetes/Chlorobi group (29.04%) and Firmicutes with only 2.55% of the bacterial reads (Figure 1). This highly imbalanced taxonomic composition between both metagenomes was also detected at lower taxonomic levels (Figure 2). In the metagenome of the lab-reared population, 83.8% of the bacterial reads were assigned to the genus *Staphylococcus*, followed in abundance by *Lactobacillus* (4.05%) and *Leuconostoc* (2.8%). This reveals a strongly imbalanced taxonomic composition with only six bacterial genera exceeding 1% of the total reads, including a massive predominance of *Staphylococcus* spp., whereas the remaining genera displayed a residual abundance. In contrast, significantly higher heterogeneity was observed in the taxonomic composition of the metagenome of the field population, with the Flavobacteria being the most abundant bacterial genus. About 12.5% of the reads in MG-rast analysis were assigned to this group, with 18 bacterial genera exceeding 1% of the total reads (Figure 2).

This heterogeneity in the taxonomic composition of each *O. nubilalis* population is also shown by the quality of the assemblies of both metagenomes and by the taxonomic profile of the predicted genes in the non-redundant gene set (Table 1). A relatively large number of sequence reads was assembled into contigs in each metagenome (99.6% in the lab population metagenome and 92.8% in the field population metagenome). However, whereas the assembly of the metagenome of the field population yielded a total of 31,928 contigs of an average length of 1.07 kb, the assembly of the metagenome of the lab population yielded only 725 contigs of an average length of 6.64 kb, with a maximum contig size of 364.2 kb (which is in the range of the complete genomes of several bacteria), as well as eight contigs with more than 100 kb.

**Table 1:** Summary of sequencing statistics of *O. nubilalis* metagenomes.

Characteristics	*O. nubilalis* lab metagenome	*O. nubilalis* field metagenome
Number of raw reads generated	437501	538480
Raw bases generated	136.42 Mb	190.32 Mb
Average read length	311.82 bp	353.45 bp
**Assembly statistics**		
—Number of assembled contigs	725	31928
—Largest contig size	364.284 Kb	60.975 Kb
—N50	54.18 Kb	1.89 Kb
—Average contig size	6.64 Kb	1.07 Kb
—Total assembled contig length	4.81 Mb	34.21 Mb
—Number of assembled reads	435770	499776
**Gene prediction results**		
—Total predicted ORFs	5203	57964
—Coding density assemblies	85.18%	82.35%
—Total non-redundant ORFs	5071	54412

In order to confirm the taxonomic binning observed in the analysis of sequence reads, the compositional profile of the assembled contigs of both metagenomes was analyzed with the TACOA program. For comparative purposes, results were extrapolated to sequence reads with the number of reads per contig from the metagenome assemblies. This analysis confirmed the massive presence of *Staphylococcus* spp. in the metagenome of the lab population, with a total number of 306,869 reads assigned (70.14% of total metagenome reads) (See Table S1). In contrast, the metagenome of the field population revealed Proteobacteria as the most abundant phylum although with a relatively low number of reads: 38,207 reads in 3808 contigs (7.1% of total metagenome reads). For 22,615 contigs, which included 350,132 reads (65% of total metagenome reads), there was no possible taxonomic classification other than at the kingdom level, with this compositional approach. This can be explained by the bias associated with small sequences of these compositional-based classification methods [10].

### Taxonomic profile of non-redundant gene sets in *O. nubilalis* metagenomes

In order to establish a non-redundant gene set for the gut microbiota of the two populations of *O. nubilalis*, the MetaGeneAnnotator program was used for gene prediction over the assemblies of sequence reads. The differences observed between assemblies of both metagenomes as a consequence of their strongly imbalanced taxonomic composition were also reflected in the number of non-redundant genes in both metagenomes (See Table 1). For the lab population, a total number of 5203 ORFs occupying 85.18% of the contigs were initially predicted, of which 5095 were longer than 100 bp. This is comparable with the value found in fully sequenced bacterial genomes (86% approximately). After removing 24 redundant ORFs by pair-wise comparisons using a very stringent criterion of 95% of identity over 90% of the length of the shorter ORF, a final set of 5071 non-redundant ORFs with an average length of 806 bp were obtained. By contrast, a total number of 57,964 ORFs were predicted in the metagenome of the field population (82.35% of the total contig length), which, after removing small and redundant ORFs, yielded a final non-redundant set of 54,412 ORFs with an average length of 524 bp, smaller than that of the non-redundant gene set of the lab population. This could be explained by the more fragmented nature of the assembly of the field metagenome as consequence of its more heterogeneous taxonomic composition.

**Figure 3:**
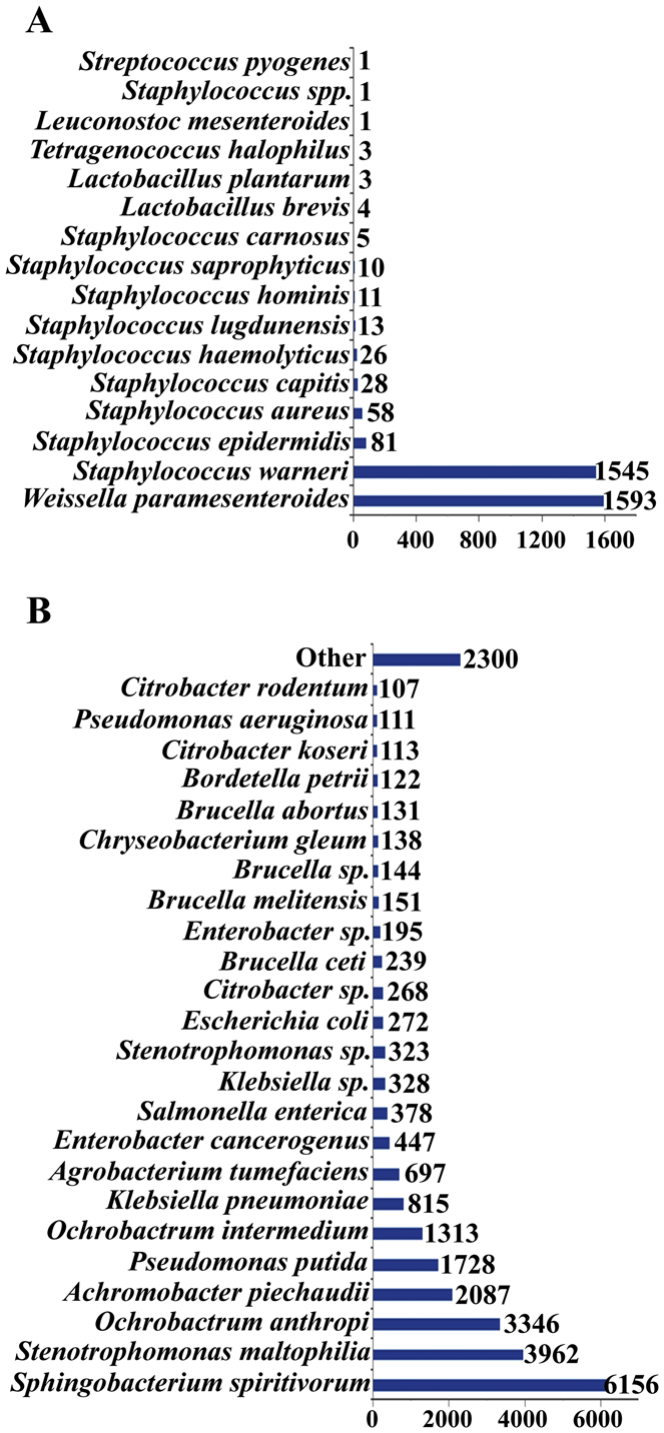
Taxonomic profiling of predicted genes in *O. nubilalis* midgut metagenomes. Taxonomic assignment of the non-redundant gene set based on best-hit on BLASTP searches over non-redundant subdivision of GeneBank microbial and viral proteins. (A) *O. nubilalis* lab metagenome; (B) *O. nubilalis* field metagenome.

To further compare the taxonomic composition of both metagenomes at the species level, an analysis of the taxonomic profile of genes from non-redundant gene sets was carried out based on BLASTP searches against an in-house database, composed by the microbial and viral subdivision of GenBank. With a restrictive threshold of 80% of identity over a minimum of 90% of the amino acid sequence of the query proteins, major differences were observed between both metagenomes in the number of species for which a significant similarity at the protein-sequence level were obtained (Figure 3). Regarding the lab-population metagenome, a total number of 3383 non-redundant ORFs gave significant similarity with proteins from the database (66.7% of the non-redundant set) belonging to 16 different bacterial species, of which ten corresponded to different *Staphylococcus* species. This is in concordance with the results of the taxonomic binning of sequence reads. However, a massive predominance of two particular species is observed, with 1593 genes assigned to the lactobacteria *W. paramesenteroides* (ATCC 33313) and 1545 genes assigned to *S. warneri* (L37603); followed in abundance by *Staphylococcus epidermidis*, with only 81 genes assigned. In contrast, a more heterogeneous taxonomic profile was observed in the metagenome of the field population, with 25,863 genes of the non-redundant gene set showing significant similarities with proteins of the database (47.5% of the non-redundant gene set) belonging to 297 different bacterial species, 24 of which had more than 100 genes assigned. The bacteroidetes *Sphingobacterium spirivorum* was the most abundant bacterial species in terms of gene content with 6156 genes of the non-redundant gene set, followed by several proteobacterial lineages such as the gamma-proteobacteria *Stenotrophomonas maltophilia* (3962 genes), the alpha-proteobacteria *Ochrobactrum anthropi* (3346 genes), or the beta-proteobacteria *Achromobacter piechaudii* (2087 genes). A complete list of the species assigned to each metagenome and their corresponding genes of the non-redundant gene set is provided in Table S2.

### Functional assignment of predicted genes and definition of common and specific gene sets

**Figure 4:**
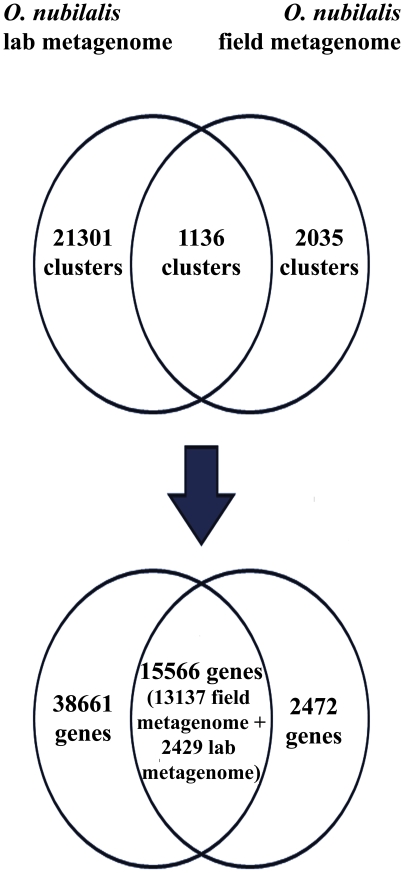
Common and population-specific gene sets in *O. nubilalis* midgut metagenomes. Venn diagram representing the results of the cluster analysis of non-redundant gene sets for each metagenome.

**Figure 5:**
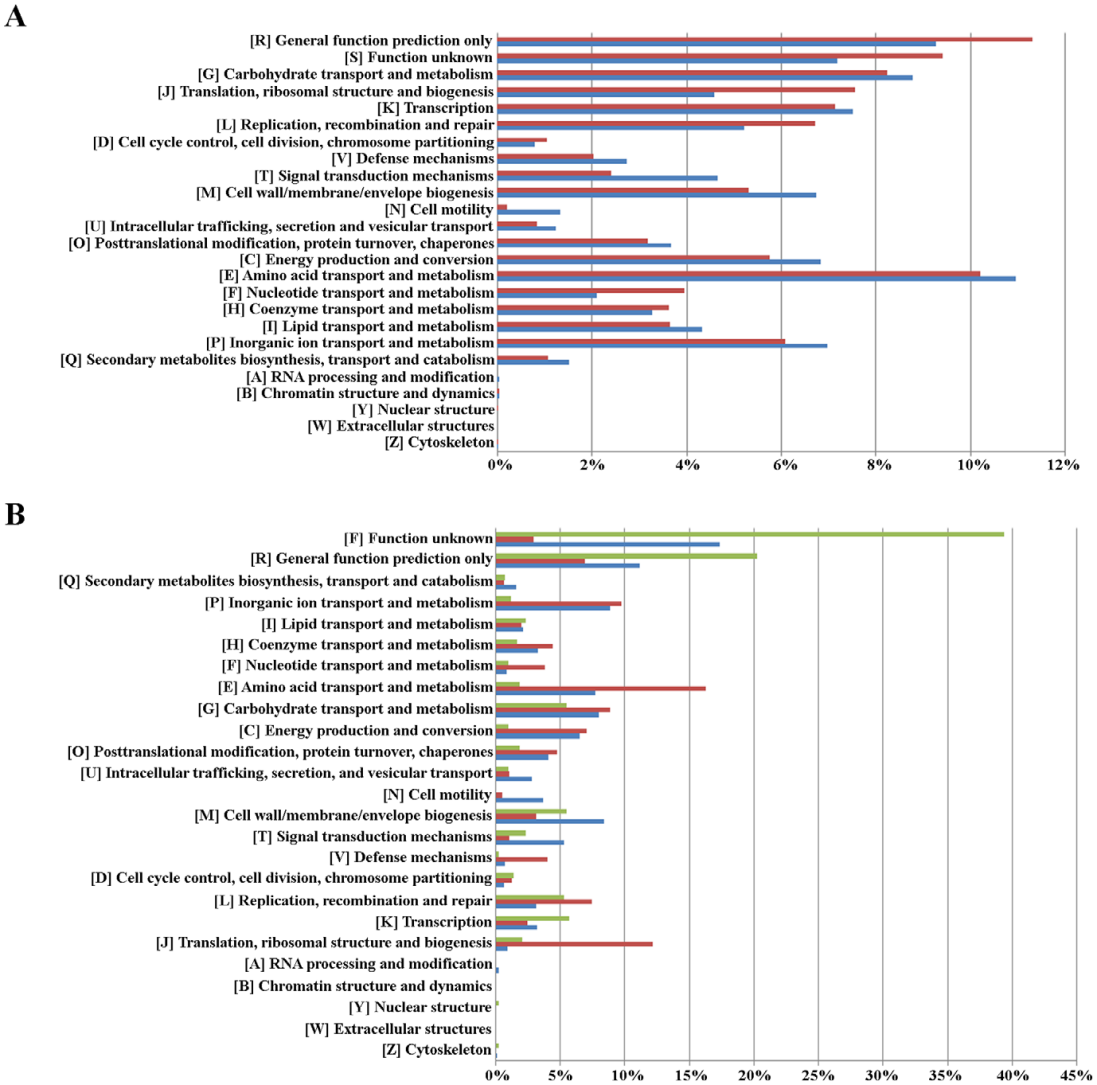
Functional characterization of non-redundant gene sets of *O. nubilalis* midgut metagenomes. (A) Bar diagram representing the distribution of COG categories in both metagenomes; (B) Bar diagram representing the distribution of COG categories in the shared and metagenome-specific gene sets defined in the cluster analysis.

The functional assignment of predicted genes in each metagenome was made on the basis of two commonly used identifiers of gene function in comparative genomic studies: the COG numbers and the KO identifiers of the KEGG database. COG assignment was based on the results of all VS of all BLASTP analysis over a dataset comprised by both non-redundant gene sets for each metagenome and the COG database available at NCBI. Cluster analysis of these results characterized gene clusters associated with well-defined functional categories based on their COG profiles (see Methods). The results of the cluster analysis are shown in Figure 4. A core set of 1136 gene clusters, including 15,566 genes from both metagenomes, was identified (13,137 genes from the field metagenome and 2429 genes from lab metagenome), of which 15,507 genes (99.6%) were assigned to 1016 COGs. In contrast, 21,301 clusters were field-specific, comprising 38,661 genes of which 15,903 genes (41%) were assigned to 2278 COGS, whereas 2035 clusters comprising 2472 genes were lab-specific, of which 1133 genes (45.8%) were assigned to 608 COGS. These results revealed major differences in gene composition between both metagenomes, with only 24% of non-redundant genes in the field metagenome and 48% of the genes in the lab metagenome being homologous at sequence level in both ECB populations. However, comparison of the profiles of COG functional categories in both metagenomes revealed very similar profiles (Figure 5A), with a large fraction of genes in both metagenomes related with amino acid transport and metabolism (11% in field metagenome and 10.2% in lab metagenome), followed by genes involved in carbohydrate metabolism (8.8% and 8.2%, respectively). Both metagenomes differed mainly in the higher frequency of genes required for translation, ribosomal biogenesis and nucleotide metabolism in the lab population metagenome; and a significant (albeit low: 1.3%) frequency of genes in the field metagenome involved in cell motility, which are almost absent in the lab-reared metagenome. In addition, a large fraction of genes in both metagenomes were associated with unknown or general functions, as deduced by the COG classification scheme (16.5% in the field metagenome and 20.7% in the lab metagenome). This, together with the large set of identified genes without functional assignment by COG or KO (1475 genes in lab metagenome and 24,411 genes in field metagenome) could indicate high levels of complexity at the gene level, which is a common feature of many metagenomic studies [11], [12].

A comparison of the functional profile of the common and metagenome-specific gene sets defined was made by cluster analysis (Figure 5B). This analysis revealed greater endowment of the common set of genes required for basic cellular functions, such as translation and ribosomal functions or amino acid metabolism, whereas a relatively large fraction of genes involved in transcription (regulators, repressors-antirepressors), cell envelope and membrane biogenesis, and specially genes with unknown or general functions, were found to be metagenome specific.

### Search for glycoside hydrolase domains and carbohydrate-binding modules

table-1-captionIn order to determine the differences between both metagenomes in terms of polysaccharide degradation activities correlated to the different nutritional sources of each ECB population, the glycoside hydrolase (GHs) domains and carbohydrate-binding modules (CBMs) were characterized following the CAZy database classification scheme. As expected from the highly imbalanced taxonomic composition, differences were found in the set of GHs and CBMs of both metagenomes. A total of 196 genes from the non-redundant gene set of the field population metagenome encoded GHs domains belonging to 32 families from the CAZy database (containing a total of 123 families). On the other hand, the lab-reared population metagenome showed a lower functional diversity with only 29 genes from the non-redundant set encoding GHs domains belonging to 12 different families from the CAZy database. All these families were shared with the field population metagenome (Table 2). The most abundant functional role in both metagenomes corresponded to the oligosaccharide-degrading enzymes: 15 GH families (47%) and 142 genes (72.4%) in the field population metagenome, whereas the lab-reared population metagenome contributed with seven families (58.3%) and 13 genes (44.8%) (Table 2). The three most populated categories in the field population metagenome accounting for 72 genes (50.7% within the oligosaccharide-degrading enzymes group) were those corresponding to exo-acting glycosidases, e.g. β-galactosidades (GH2 with 28 genes), α-L-fucosidase (GH29 with 21 genes) and α-mannosidase (GH92 with 23 genes). Of those GHs families only GH2 was represented in the metagenome of the lab-reared population with three genes (23%). Remarkably, typical processive cellulases were represented by only the family GH5 with two genes (1%) in the field population metagenome whereas the lab-reared population metagenome showed none (Table 2). Endohemicellulases were represented by five families (15.6%) and 12 genes (6.1%) in the field population metagenome and by only one family (8.3%) and two genes (6.9%) in the lab-reared population metagenome (Table 2). The amylase functional category was the only GH family containing more genes in the lab metagenome than in the field metagenome, with six genes (20.6%) in the former and five (2.6%) in the latter (Table 2). Regarding CBMs distribution, 25 (out of a total of 61 in the CAZy database) domains were identified in the field population metagenome distributed in 91 genes (Table 3). Some of these modules are in accordance to the GHs detected (e.g. CBM38 and CBM58). In the lab-reared population metagenome four CBMs were detected in 27 genes. The most populated category (CBM50) corresponded to carbohydrate-binding domains of enzymes involved in bacterial cell wall remodeling. Again the only the CBM category with more genes in the lab-reared population metagenome than in the field metagenome, was that corresponding to amylases (CBM58) (Table 3).

**Table 2:** Putative glycoside hydrolases (GH) counts in the metagenomes of *O. nubilalis* field and lab populations.

CAZy family*		Known activities	Correlated PFAM domain	Field metagenome †	Lab metagenome †
**Cellulases**	GH5	Chitosanase, β-mannosidase, cellulase, glucan 1,3-β-glucosidase, and others	Cellulase	2	0
**Endohemicellulases**	GH10	Endo-1,4-β-xylanase, endo-1,3-β-xylanase	Glyco_hydro_10	4	0
	GH26	β-mannanase, β-1,3-xylanase	Glyco_hydro_26	1	0
	GH28	Polygalacturonase, exo-polygalacturonase, rhamnogalacturonase, and others	Glyco_hydro_28	2	0
	GH30	Glucosylceramidase, β-1,6-glucanase, β-xylosidase	Glyco_hydro_30	4	0
	GH32	Endo-inulinase, endo-levanase, exo-inulinase, and others	Glyco_hydro_32N	1	2
**Debranching enzymes**	GH78	α-L-rhamnosidase	Bac_rhamnosid	5	0
	GH51	α–L-arabinofuranosidase	Alpha-L-AF-C	5	1
**Amylases**	GH13	α-amylase, pullulanase, cyclomaltodextrin glucanotransferase, and others	Alpha-amylase	4	6
	GH15	Glucoamylase, glucodextranase, α-trehalase	Glyco_hydro_15	1	0
**Oligosaccharide-degrading enzymes**	GH1	β-glucosidase, β-galactosidase, β-mannosidase, and others	Glyco_hydro_1	2	5
	GH2	β-galactosidase, β-mannosidase, β-glucuronidase, and others	Glyco_hydro_2	28	3
	GH3	β-glucosidase, xylan 1,4-β-xylosidase, β-N-acetylhexosaminidase, and others	Glyco_hydro_3	13	1
	GH4	Maltose-6-phosphate glucosidase, α-glucosidase, α-galactosidase, and others	Glyco_hydro_4	3	0
	GH20	β-hexosaminidase, lacto-N-biosidase, β-1,6-N-acetylglucosaminidase, and others	Glyco_hydro_20	13	0
	GH27	α-galactosidase, α-N-acetylgalactosaminidase, isomalto dextranase, and others	Melibiase	1	1
	GH29	α-L-fucosidase	Alpha_L_fucos	21	0
	GH31	α-glucosidase, α-1,3-glucosidase, sucrase-isomaltase, and others	Glyco_hydro_31	4	0
	GH37	α,α-trehalase	Trehalase	2	0
	GH42	β-galactosidase	Glyco_hydro_42	1	1
	GH65	Maltose phosphorylase, trehalose phosphorylase, and others	Glyco_hydro_65m	1	1
	GH88	d-4,5 unsaturated β-glucuronyl hydrolase	Glyco_hydro_88	10	0
	GH43	Arabinases and xylosidases	Glyco_hydro_43	18	1
	GH92	α-mannosidase	Glyco_hydro_92	23	0
	GH97	α-glucosidase, α-galactosidase	Glyco_hydro_97	2	0
**Chitinases**	GH18	Chitinase, endo-β-N-acetylglucosaminidase	Glyco_hydro_18	5	0
**Cell wall remodelling**	GH16	Xyloglucan, keratan-sulfate endo-1,4-β-galactosidase, endo-1,3-β-glucanase, and others	Glyco_hydro_16	2	0
	GH24	Lysozyme	Phage_lysozyme	5	0
	GH25	Lysozyme	Glyco_hydro_25	2	5
	GH102	Peptidoglycan lytic transglycosylase	MltA	2	0
	GH104	Peptidoglycan lytic transglycosylase	Phage_lysozyme	5	0
	GH73	endo-β-N-acetylglucosaminidase	Glucosaminidase	4	2

† Number of detected ORFs in each GH family in non-redundant gene sets of *O. nubilalis* lab and field metagenomes.

*GHs are grouped according to major functional role (cfr. [26]).

**Table 3:** Putative carbohydrate-binding modules (CBM) in the metagenomes of field and lab *O. nubilalis* populations.

CBM family	Known activities	Correlated PFAM domain	Field metagenome†	Lab metagenome†
CBM4	Xylan-, glucan-, and amorphous cellulose-binding domains	CBM_4_9	2	0
CBM9			2	0
CBM16			2	0
CBM22			2	0
CBM37			2	0
CBM54			2	0
CBM5	Carbohydrate binding in	CBM_5_12	1	0
CBM12	glycosyl hydrolase enzymes		1	0
CBM6	Amorphous cellulose- and xylan-binding domain	CBM_6	2	0
CBM35			2	0
CBM36			2	0
CBM56			2	0
CBM13	Xylan-, mannose, and galactose residues binding domain	Ricin_B_lectin	4	0
CBM32	Polygalacturonic acid-, galactose- and lactose-binding domain	F5_F8_type_C	17	0
CBM33	Chitin-binding domain	Chitin_bind_3	2	0
CBM38	N-terminal domain of GH32	Glyco_hydro_32N	1	2
	C-terminal domain of GH32	Glyco_hydro_32C	1	2
CBM41	Amylose-, amylopectin-, pullulan-	CBM_48	7	0
CBM48	and glycogen-binding domains (associated to GH13)		7	0
CBM46	C-terminal domain of GH5, and	Cellulase	2	0
CBM59	mannan-, xylan-, and cellulose-binding domain		2	0
CBM50	Peptidoglycan-binding domain in enzymes involved in bacterial cell wall degradation	LysM	16	17
CBM51	Galactose-binding domain in several GHs	NPCBM	1	0
CBM57	Di-glucose-binding domain	Malectin	1	0
CBM58	Active site domain of GH13	Alpha-amylase	4	6
CBM61	α-galactosidases of GH31	Glyco_hydro_31	4	0

† Number of detected ORFs in each CBM family in non-redundant gene sets of *O. nubilalis* lab and field metagenomes.

### Genome assembly of the two predominant bacteria in lab population metagenome

The quality of the assembly of the sequence reads from the lab population metagenome, with 99.6% of the sequences assembled in 725 contigs with many having sizes higher than 50 kb, and the results of the taxonomic profiling of genes from the non-redundant set, with 94.7% of genes with significant similarity (RefSeq analysis) to *S. warneri* (1545 genes) and *W. paramesenteroides* (1593 genes) suggested that the complete genomes of these two bacterial species were nearly complete in the metagenome of the lab population. In order to confirm this hypothesis, a comparative genomics approach was carried out by comparing the set of contigs of both bacteria with the reference genome assemblies of *S. warneri* L37603 (NZ_ACPZ00000000) and *W. paramesenteroides* ATCC 33313 (NZ_ACKU00000000). A total of 52 contigs had genes assigned to *S. warneri*, including the eight contigs with more than 100 kb of the assembly, whereas a total number of 143 contigs included genes assigned to *W. paramesenteroides*, with the largest contig measuring 96.6 kb in size. After filtering those contigs without significant similarity at whole sequence level with their corresponding reference genomes through TBLASTX searches following ACT visualization, 35 contigs from *S. warneri* and 125 contigs from *W. paramesenteroides* showed significant similarity with their corresponding reference genomes. The sum of the length of all these significant contigs gave an estimated genome length of 1.899 Mb for *W. paramesenteroides* and 2.46 Mb for *S. warneri*, which is very similar to the predicted length of *S. warneri* L37603 (2.425 Mb) and *W. paramesenteroides* ATCC 33313 (1.962 Mb) genomes, indicating that both genomes were indeed almost complete in the metagenome of the lab-reared ECB population. This was further confirmed when the whole set of contigs from each bacterium was reordered based on the reference scaffold of the whole set of contigs from each reference genome with MAUVE. As a result, most of the reference genomes appeared covered by contigs from the lab-reared metagenome with high levels of nucleotidic identity, especially for contigs corresponding to *W. paramesenteroides* (Figure S1A). In contrast, contigs corresponding to *S. warneri* displayed low levels of nucleotidic identity with the reference genome of *S. warneri* L37603, as well as a higher rearranged structure in terms of synteny conservation between contigs (Figure S1B). Phylogenetic analysis with the only two complete genes in the lab metagenome corresponding to ribosomal 16S confirmed the taxonomic assignment of *W. paramesenteroides* at the species level, whereas the other complete 16S genes, although with more than 97% of identity with *S. warneri* L37603, were closer to *Staphylococcus pasteuri* (ATCC51129T), with *S. warneri* (L37603) as common outgroup of both species (See Figure S2). This explains the lower levels of nucleotidic identity of *S. warneri* contigs with the reference genome of L37603 strain, with *S. pasteuri* being the closest relative to the *Staphylococcus* strain of the metagenome. This would not be detected with other taxonomic binning approaches due to the absence of sequences of this taxon in public databases, apart from 16S sequences.

**Figure 6:**
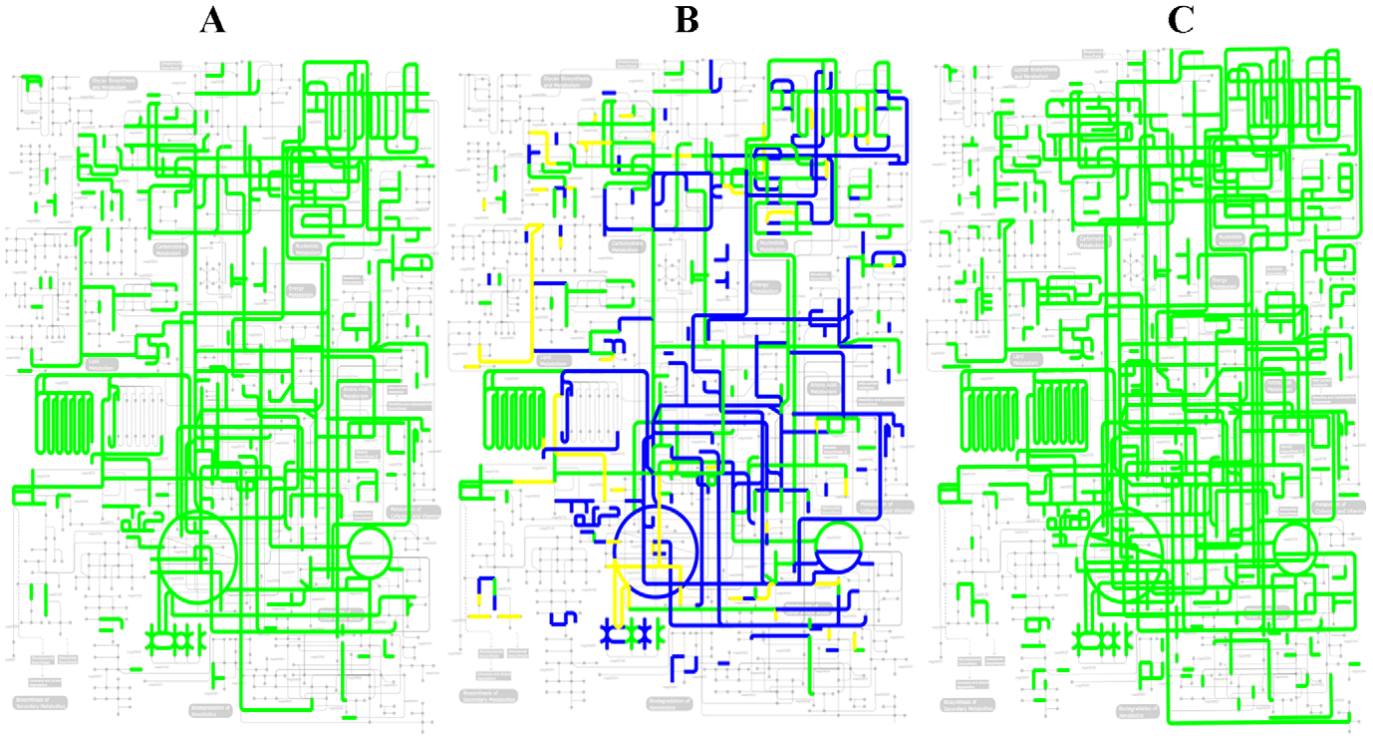
Metabolic maps of non-redundant gene sets. Projection of the KO identifiers of non-redundant gene sets of each metagenome assigned with KAAS annotation server on KEGG pathway maps using the iPath tool. (A) Metabolic map of lab population metagenome; (B) Metabolic map of lab population metagenome inferred from *S. warneri* and *W. paramesenteroides* genes (blue = *S. warneri* specific KO; green = Common KO *S. warneri*-*W. paramesenteroides* genes; yellow = *W. paramesenteroides* specific KO); (C) Metabolic map of field population metagenome.

**Figure 7:**
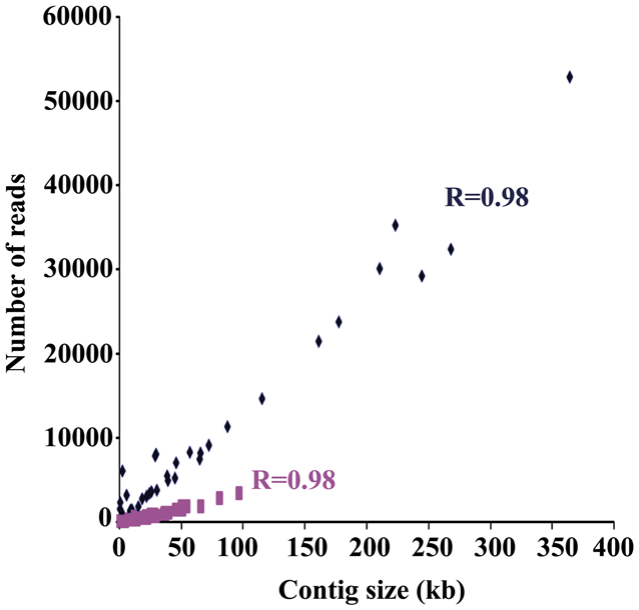
Contig size VS. read number in the *O. nubilalis* lab metagenome sequence assembly. Correlation between contig size and read number in *S. warneri* (blue) and *W. paramesenteroides* (red) contigs from the assembly of lab population metagenome. The correlation coefficient (r) for each contig set is reported.

Finally, additional evidence about the near completeness of these two genome sequences came from the reconstruction of the metabolic map of the whole metagenome (See Figure 6). When the taxonomic profile of the genes from the non-redundant gene set was considered in the metabolic map, most of the well-defined metabolic functions and pathways were exclusively associated with *S. warneri* and *W. paramesenteroides* genes. This includes genes encoding the complete ribosomal machinery and genes for the whole set of aminoacyl tRNA synthetases, which are considered evidence of the near-completeness of genome sequences despite their subdivision in several contigs [13]. *S. warneri* was associated with a 43.6× coverage and that of *W. paramesenteroides* was of 8.8×. This was a consequence of major differences in the number of reads of both genomes. This was also concluded when taxonomic profiling of contig assemblies was considered in the correlation between contig size and number of reads (Figure 7). This difference in the relative abundance of both main bacterial species of the lab-reared sample was also obtained when the community structure of the metagenome is inferred from the BLASTN profiles of the reads against an internal database of complete bacterial and viral genomes that include the draft assemblies of *S. warneri* L37603 and *W. paramesenteroides* ATCC 33313 (See Figure S3). *S. warneri* L37603 was the predominant taxon with a relative abundance of 49.2% of the metagenomic reads, followed by *W. paramesenteroides* ATCC 33313 (27% of the metagenome reads) and by the *Staphylococcus* phage PT1028 (11.6% of the metagenome reads). The remaining bacterial species had a minor prevalence in the lab-reared microbiota, with relative abundances lower than 1%.

## Discussion

In the present study we aimed to compare, with high-throughput sequencing, the midgut microbiota of two populations of the ECB. Since antibiotics dramatically affect the composition of intestinal microbiota in Lepidoptera [14], we used a preservative-free diet, which was changed every day in order to avoid moisture accumulation. It should be noted, however, that the midgut microbiota of the lab-reared population had been exposed to antiseptic compounds during rearing prior to our study, and it is therefore likely that the microbiota was consequently altered compared to the original starter population. Indeed, a recent report [7] describing a range of antibiotics resistance determinants in gypsy moth suggests that lab-reared insect guts are a reservoir of antibiotic resistance genes with the potential for dissemination.

There are very few reports comparing diet-dependent biological diversity of the insect midgut. A comparison of diet-fed and naturally occurring caterpillar populations has previously been reported for *Helicoverpa armigera* [15], showing that the laboratory population harbored a rather simple gut microbiota consisting mostly of phylotypes belonging to Enterococcus (84%). For the field population, the authors reported than phylotypes belonging to Enterococcus (28%) and Lactococcus (11%), as well as Flavobacterium (10%), Acinetobacter (19%), and Stenotrophomonas (10%) were dominant members. Low diversity has been also reported, using both culture and culture-independent methods, in another Lepidoptera, *Lymantria dispar* [16]. Also, another species of corn borer, *Sesamia nonagrioides* has been previously studied, but the study was restricted to trypsins, a relatively high diversity of which was found [17]. The midgut of the cabbage white butterfly was studied using 16S ribosomal RNA gene sequences to describe variation among bacterial communities and the observed species richness ranged from only six to 15, depending on diet and time variables, and the most abundant members affiliated with the genera *Methylobacteria*, *Asaia*, *Acinetobacter*, *Enterobacter*, and *Pantoea* [18]. Finally, a high-throughput study was performed on artificially reared gypsy moth by Guan and coworkers [19]. In concordance with our work, a *Staphylococcus*-rich microbiota was reported; however, the overall diversity of the midgut ecosystem was not described.

Our results are the first complete metagenomic characterization of a lepidopterous insect and reveal that the wild ECB population is mainly associated with Proteobacteria, and bacteroidetes/Chlorobi; whereas the lab population is very rich in Firmicutes with six genera of ubiquous, commensal or saprofitic gram positive bacteria accounting for almost 95% of the assigned reads. The abundance of two of these bacteria: *W. paramesenteroides* and *S. warneri* was so high that reordering of the contigs allowed a draft sequence of their whole genomes, which we present here. This extraordinary imbalance in bacterial populations may be a consequence of the multiplication of generalist commensal bacteria in the artificial diet prior to insect feeding, and it is in contrast with the much more balanced distribution of gram-negative bacterial species isolated from a range of ecological niches detected in the field metagenome. Several of the most frequently represented genera identified in this sample (*Bacteroides*, *Pseudomonas*, *Cytophaga*, *Bordetella*, etc) are well-known cellulase producers [20], [21], [22], [23], while others are members of insect-associated flora (*Klebsiella*, *Enterobacter*), or are known to be associated with plant surfaces (*Agrobacterium*, *Mesorhizobium*, *Rhizobium*, etc). Taken together, these results suggest a clear association between the environment (artificial rearing versus on-crop natural populations) and the composition of the main bacterial taxa in the ECB midgut.

Our results reveal the highest microbial diversity in a Lepidoptera midgut reported to date: we identified about 240 genera in *O. nubilalis*. This is in contrast with previous reports describing species richness of a few dozens of bacterial species at most. Many of the taxa detected were found in both the lab-reared and wild (field) collections while taxonomic binning of both metagenomes reveals that a core of species of around 200 different genera is present in the ECB midgut regardless of whether the insects feed on artificial starch-rich diet or on cellulose-rich crops. The existence of such a vast core of shared taxa between both metagenomes is however compatible with the highly imbalanced biodiversity revealed by the taxonomic assignment over the non-redundant gene set (Fig. 3). In other words, both metagenomes differ greatly regarding the relative proportion of the most frequent bacteria harbored, but are similar in terms of total taxonomic composition, with many of the taxa being present at a very low (less than 1%) proportion. Although the enrichment step on solid media did not prevent detection of tissue-associated, typically unculturable, bacteria such as *Sodalis* spp., this step might certainly imply a bias in the relative proportion of culturable versus unculturable bacteria. However, this bias certainly does not underlie the differences detected between both metagenomes, since the enrichment steps and isolation conditions were identical in both cases.

The functional profile of non-redundant gene sets in both metagenomes also shows hallmarks of this imbalanced composition of both metagenomes with respect to the most prevalent taxa. When the total number of unique COG categories identified in both metagenomes is compared with that of other metagenomes, 1602 unique COGs were identified in the metagenome of the lab population, similar to that described for the acid mine drainage metagenome (1824 COGs), for which two predominant species were also identified whose genomes were almost completely sequenced in a metagenomic survey [13]. In contrast, 2886 unique COGs were identified in the field population metagenome, which are closer to more complex metagenomes like that of soil (3394) or the classical example of the Sargasso Sea samples [11]. The cluster analysis of non-redundant gene sets also reveals that only a minor fraction of genes have corresponding homologs in both metagenomes (24% in the field metagenome and 48% in the lab metagenome), mainly associated with basic cellular functions like translation, informational structure and biogenesis or aminoacid transport and metabolism. In contrast, most of the genes specific of each metagenome correspond to genes of unknown or general function, although functional classes related with environmental information processing, like cell envelope structure and biogenesis or signal transduction mechanisms, also have a large fraction of metagenome-specific genes compared to homologous genes in both metagenomes. This fact can be explained by the different environment associated with each *O. nubilalis* population. However, a similar functional profile is observed when the distributions of non-redundant genes in different functional categories are compared between metagenomes (see Figure 5A), revealing that although the metagenomes differ from each other within particular functional categories related to the different environmental conditions of each *O. nubilalis* population, the distribution between functional categories is rather similar in both metagenomes.

Lab-reared and field-collected ECB also differ significantly in terms of their GH and CBM profiles. As expected, the field population displayed a much larger set of hydrolytic enzymes, particularly oligosaccharide-degrading activities and CBMs related to insoluble carbohydrate-binding (e.g., CBM37, CBM54). This is consistent with the existence of a strong selection pressure in the field population favoring bacterial enzymatic activities for the degradation of plant complex polysaccharides. This selection pressure was absent in the lab population, which was fed with simpler polysaccharides, mainly starch, as reflected by the relative abundance of GHs and CBMs related to amylases in the corresponding metagenome. Compared with other lignocellulosic-degrading microbiomes, both samples showed a striking scarcity of canonical cellulases. The family GH5, characteristic of processive cellulases, appears to be a minor component of the enzymatic machinery since it is the only one identified in the field population metagenome (but not in the lab-reared population sample). This GH family has been universally identified in the metagenomes of the bovine gut [24], termite hindgut [4], Tammar wallaby foregut [25], as well as in many cellulolytic bacterial communities [26]. Nevertheless, our sample derived from the field population showed a diversity of GH catalytic domains and genes in the functional category of hemicellulases, involved in the degradation of internal bonds in cellulose and other polysaccharides like xylan or mannan [27], as well as a good representation of the corresponding CBMs (e.g. the PFAM domains CBM_4_9, CBM_6, and Ricin_B_lectin). However, the highest abundance of families and genes were observed in the oligosaccharide-degrading category (Table 2), also involved in the hydrolysis of noncellulosic polysaccharides and the side chains of hemicelluloses and pectins [27]. This relative abundance of activities other than hydrolysis of the main chain of cellulose has also been observed in bovine gut metagenomes [24], in contrast to the termite hindgut microbiome [4]. These correlations are concordant with the chemical composition of the *O. nubilalis* field population's diet (pepper leaves), which more closely resembles that of ruminants (forages) than termite's (wood). As in the case of ruminants, the microbiome enzymes in *O. nubilalis* show a preference for the easily available side chains of complex plant polysaccharides [3]. This case of remarkable functional convergence can also be observed in the plant biomass-degrading abilities of the external microbial community in the fungus garden of the leaf-cutter ants [28].

From our results it cannot be concluded whether the unusually high bacterial diversity of the ECB midgut is a rare attribute of the tested species or rather a consequence of the high-throughput methodology used. In fact, the species richness of ECB might actually be significantly higher than our estimates, since amplification on solid media is expected to reduce the number of uncultured microorganisms in the processed sample. Since most previous reports on Lepidoptera-associated flora did not use high-throughput sequencing techniques, and considering that a large majority of identified sequences were present at very low (1%) frequencies, it is likely that the potency of metagenomics lies behind the high diversity found in the *O. nubilalis* midgut. It is therefore tempting to hypothesize that the application of similar (enrichment plus pyrosequencing) strategies to other insect midguts would result in a dramatic increase in the insect-associated microbial diversity count.

## Materials and Methods

### Insects

Lab-reared insects used in this work were reared in the laboratory with a temperature of 26±1°C, a 16∶8 (L∶D) photoperiod and 65% RH. *O. nubilalis* larvae were fed on an artificial diet, reported previously [29], but with modifications involving the removal of bacteriostatic compounds such as nipagin and formol. One liter of diet was prepared as follows: corn flower (128 g), casein (14 g), beer leavening flakes (34 g) and wheat germ (32 g) were briefly cooked in sterile water. The mixture was added to an agar solution up to 1.8% final concentration and cooled to 40°C before adding 9 g vitamin complex (Sigma Aldrich), 4.5 g of ascorbic acid and 5 ml of olive oil. The second population was collected from infested red pepper greenhouses in the vicinity of Pilar de la Horadada (Alicante, Spain) in August 2009. Third- to last-instar larvae were transported to the laboratory in plastic boxes with pepper tissues as the sole food source and kept at room temperature prior to dissection.

### Gut dissection

About one hundred third- to last-instar larvae from each of the insect populations were immobilized by placing them on ice and the midgut was dissected in sterile conditions. Guts were disaggregated and manually homogenized with an Eppendorf-adapted pestle and directly inoculated onto agar plates containing suitable media as described below.

### Amplification of the microbiota on solid media

In order to increase the microbial titer, an amplification step on solid media was achieved. Homogenized midguts were spread on standard (LB) and selective media with CMC as the sole carbon source (CMC composition: 2.0 g NaNO_3_, 1 g K_2_HPO_4_, 0.5 g KCl, 0.26 g peptone, 0.25 g MgSO_4_, and 2.0 g carboxymethylcellulose per L). The pH of the CMC plates was adjusted to a range of 8 to 12. One set of inoculated LB and CMC plates was incubated at 30°C and a replica at 37°C for one to three days. The biomass corresponding to the microbiota and the midgut debris on the plates was collected with a sterile Digralsky spreader and pooled.

### DNA extraction

Total DNA was extracted with the GenElute Bacterial Genomic DNA kit (Sigma) with the Gram+ optional treatments performed as recommended by the manufacturer. The quantity and quality of the DNA was determined on 1.5% agarose gel and with a Nanodrop spectrophotometer, and then pyrosequenced.

### DNA sequencing, assembly and gene prediction

Total DNA from each population of *O. nubilalis* was used to generate a shotgun library which was then sequenced using half pyrosequencing plate on a Roche 454 FLS GS Titanium sequencer at Lifesequencing S.L. Assembly of the sequences from each population was performed using the 454 De Novo Assembler software NEWBLER (454 LifeSciences Roche) with default parameters. Table 1 summarizes the pyrosequencing results for each sample and statistical coverage. All sequences have been deposited in GenBank (accession numbers: lab-reared metagenome, SRR172996.2; field metagenome, SRR173463.1).

The MetaGeneAnnotator program [30], which is based on Hidden Markov Model (HMM) that incorporates statistical models of phage genes and Ribosomal Binding Sites (RBS) for precise prediction of transcription start sites, was employed to predict potential protein-coding regions (Open Reading Frames, ORFs) from the NEWBLER assemblies of each population.

### Construction of non-redundant gene set

An initial filtering based on sequence length was applied, and ORFs with less than 100 bp were filtered out. The remaining predicted ORFs in each metagenome were aligned to each other using BLAT [31], and gene pairs with identities greater than 95% and with an aligned coverage of over 90% of the shortest ORFs were grouped together. These groups sharing genes were then merged and the longest ORF was used to represent the group, considering the remaining ORFs as redundant. Predicted ORFs of the non-redundant gene set for each metagenome were translated into protein sequences with the program TRANSEQ from the EMBOSS package [32].

### Metagenome phylogenetic binning

The complete sets of reads and assembled contigs from each population of *O. nubilalis* were phylogenetically binned, by combining different compositional-based methods and sequence similarity-based methods. First, each metagenome was analyzed with the MG-RAST server, which is based on the SEED framework for comparative genomics [33], enabling reads to be classified based on sequence similarity searches against several internal databases. An expected e-value lower than 1×E^−5^ was used as cutoff for the BLASTX searches over the SEED non-redundant protein database. Second, the GAAS program [34] was used to determine the community composition of both metagenomes based on BLASTN searches [35] of sequence reads against a local database of 1942 complete viral, microbial, and eukaryotic genome sequences. BLASTN results were filtered by using minimum percentage of nucleotidic identity of 80%, minimum alignment coverage of 80% of the query sequence lengths, and maximum e-value cutoff of 1×E^−**5**^. Third, assembled contigs from each metagenome were classified taxonomically with the TACOA software [10], a composition-based classifier using similarity profiles between the Genome Feature Vectors (GFVs) of query sequences and a reference database of all complete prokaryotic genomes available on the KEGG database as of August 2009. Fourth, ORFs from the non-redundant gene sets of each metagenome were compared against a local database containing all microbial and viral proteomes available at the RefSeq subdivision of GenBank database as of July 2010 through BLASTP, with filtering by e-value (1×E^−5^), alignment identity (min 80%), and alignment length (min 90% query length). The taxonomic identity of the top hit was recorded.

### COG assignment and functional annotation of *O. nubilalis* metagenomes

In order to make COG assignments to ORFs from the non-redundant set of each metagenome, the reference dataset of COG (Clusters of Orthologous Groups of proteins) from the NCBI ftp site were downloaded (ftp://ftp.ncbi.nih.gov/pub/COG/COG/). A single multi-fasta file was created with non-redundant sets of ORFs from each metagenome and the reference COG dataset, and all-against-all BLASTP comparisons were carried out [35]. Results were filtered by using a maximum e-value of 1×10^−5^. The gene products selected were subsequently clustered with the program TribeMCL [36] using an inflation value of 2.5 for the clustering step, and the clusters that included genes from the reference COG dataset and the non-redundant gene set of each metagenome were subsequently analyzed for COG assignment to metagenome ORFs, as deduced by the corresponding COG of the genes from the reference dataset.

Functional annotation of the ORFs from the non-redundant set of each metagenome was carried out with the program BLAST2GO [37] based on BLASTP searches against non-redundant protein subdivision of GenBank. Metabolic maps of each metagenome were obtained with the program KAAS through BLASTP searches against the KEGG GENES database with the non-redundant sets of each metagenome followed by KO assignment by Single-Directional Best Hit method [38]. The results of the functional annotation of the non-redundant gene set characterized in each metagenome are provided in Table S3 and Table S4.

### Identification of glycoside hydrolases

Glycoside hydrolases (GHs) catalytic domains with polysaccharide degradation ability and Carbohydrate Binding Modules (CBMs) were identified on the non-redundant gene set of each metagenome by using the functional classification of the Carbohydrate Active Enzyme (CAZy) database [39] as described below.

PFAM domains associated to the different GHs and CBMs families of CAZy database were identified based on CAZy family annotations when available, and from sequence similarity searches with individual protein sequences for those families with no PFAM annotations in CAZy database. The program HMMER [40] was used to identify these PFAM domains in the protein sequences of the non-redundant gene set of each metagenome by using an maximum e-value cutoff of 10^−5^.

### Draft genome assembly of *Staphylococcus warneri* and *Weissella paramesenteroides*

TBLASTX comparisons between contigs classified as *Staphylococcus spp.* and *Leuconostoc spp.* by TACOA in the midgut metagenome of lab-reared insects was carried out against the draft assemblies of the genomes of *S. warneri* L37603 (NZ_ACPZ00000000) and *W. paramesenteroides* ATCC 33313 (NZ_ACKU00000000). Results were visualized with the Artemis Comparison Tool [41] in order to filter out those contigs that did not conserve significant similarity at whole contig length. These significant contigs were reordered with the program MAUVE [42] based on their corresponding reference genomes in order to evaluate the degree of completion of each genome in the lab-reared metagenome.

Phylogenetic analyses with the two complete 16S genes identified in the lab-reared metagenome were carried out in order to confirm the taxonomic assignment of *Staphylococcus* spp. and *Leuconostoc* spp. at the sequence level. The 16S sequences of 32 additional Firmicutes were retrieved from the RDP database (http://rdp.cme.msu.edu/). Sequences were aligned with the R-COFFEE software with default options [43], and the phylogenetic tree was reconstructed by maximum likelihood with the PHYML program and GTR model of sequence evolution. Heterogeneity in the evolution rates were assumed following gamma distribution, with four different rates estimated from the sequence alignment [44].

## Supporting Information

Figure S1 Reordering of *S. warneri* and *W. paramesenteroides* contigs from the metagenome of lab population based on the sequences of their reference genomes with MAUVE. (A) *W. paramesenteroides* ATCC 33313 (top) VS *W. paramesenteroides* contigs; (B) *S. warneri* L37603 (top) VS *S. warneri* contigs. The height of the colored lines in the collinear blocks represents the nucleotidic identity between both sequences.
(TIF)

Figure S2 Phylogenetic reconstruction of 34 complete 16S genes including the two complete 16S identified in the metagenome of the lab population (contig00689_*Ostrinia nubilalis* and contig00725_*Ostrinia nubilalis*). For details of the phylogenetic reconstruction method see Materials and Methods section.
(TIF)

Figure S3 Genome relative abundance of lab and field *O. nubilalis* metagenomes based on the GAAS program results [34].
(TIF)

Table S1 Taxonomic binning of *O. nubilalis* lab and field metagenomes based on compositional features of sequence assemblies.
(XLS)

Table S2 Number of genes assigned to different bacterial species in both metagenomes based on BLASTP best hits.
(XLS)

Table S3 Complete non-redundant gene sets predicted in *O. nubilalis* lab metagenome with their corresponding annotation.
(XLS)

Table S4 Complete non-redundant gene sets predicted in *O. nubilalis* field metagenome with their corresponding annotation.
(XLS)
