# Supplementary figures and images for: Microbial Diversity in the Midguts of Field and Lab-Reared Populations of the European Corn Borer Ostrinia nubilalis
Source: PLoS One. 2011 Jun 30;6(6):e21751. doi: 10.1371/journal.pone.0021751 (PMC3128089; doi:10.1371/journal.pone.0021751)

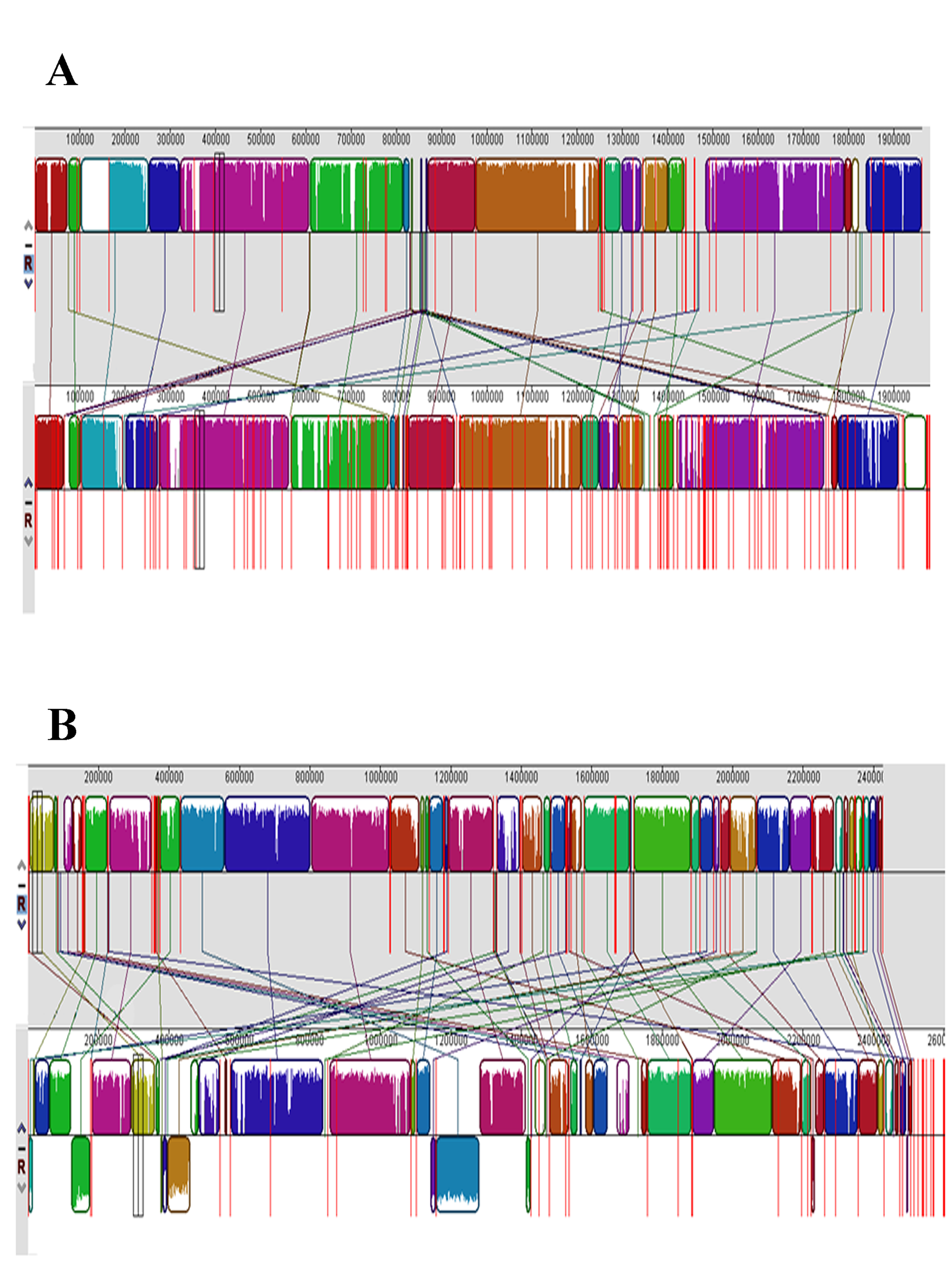

Supplement: Figure S1 — Reordering of S. warneri and W. paramesenteroides contigs from the metagenome of lab population based on the sequences of their reference genomes with MAUVE. (A) W. paramesenteroides ATCC 33313 (top) VS W. paramesenteroides contigs; (B) S. warneri L37603 (top) VS S. warneri contigs. The height of the colored lines in the collinear blocks represents the nucleotidic identity between both sequences. (TIF) [file pone.0021751.s001.tif]

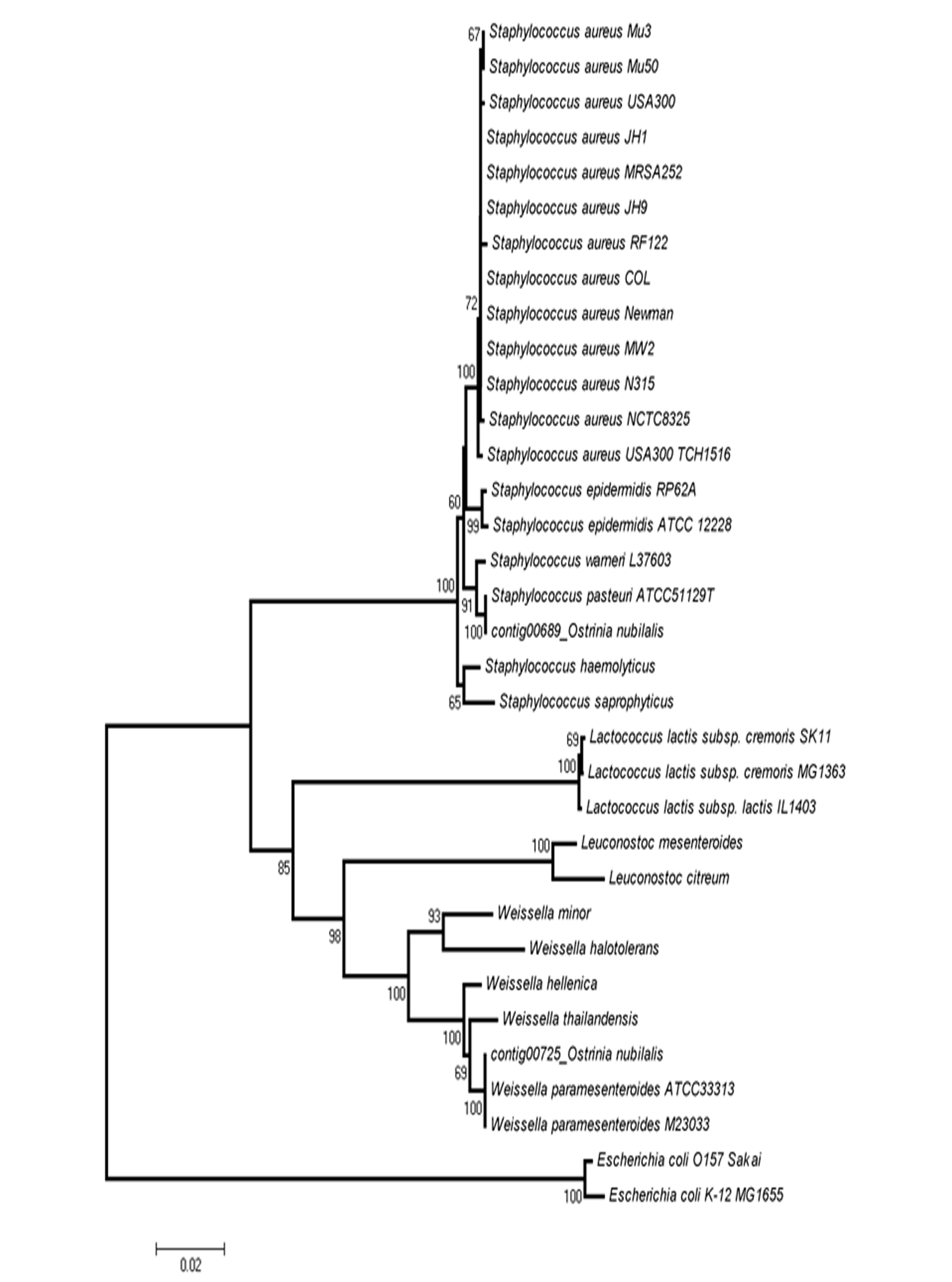

Supplement: Figure S2 — Phylogenetic reconstruction of 34 complete 16S genes including the two complete 16S identified in the metagenome of the lab population (contig00689_Ostrinia nubilalis and contig00725_Ostrinia nubilalis). For details of the phylogenetic reconstruction method see Materials and Methods section. (TIF) [file pone.0021751.s002.tif]

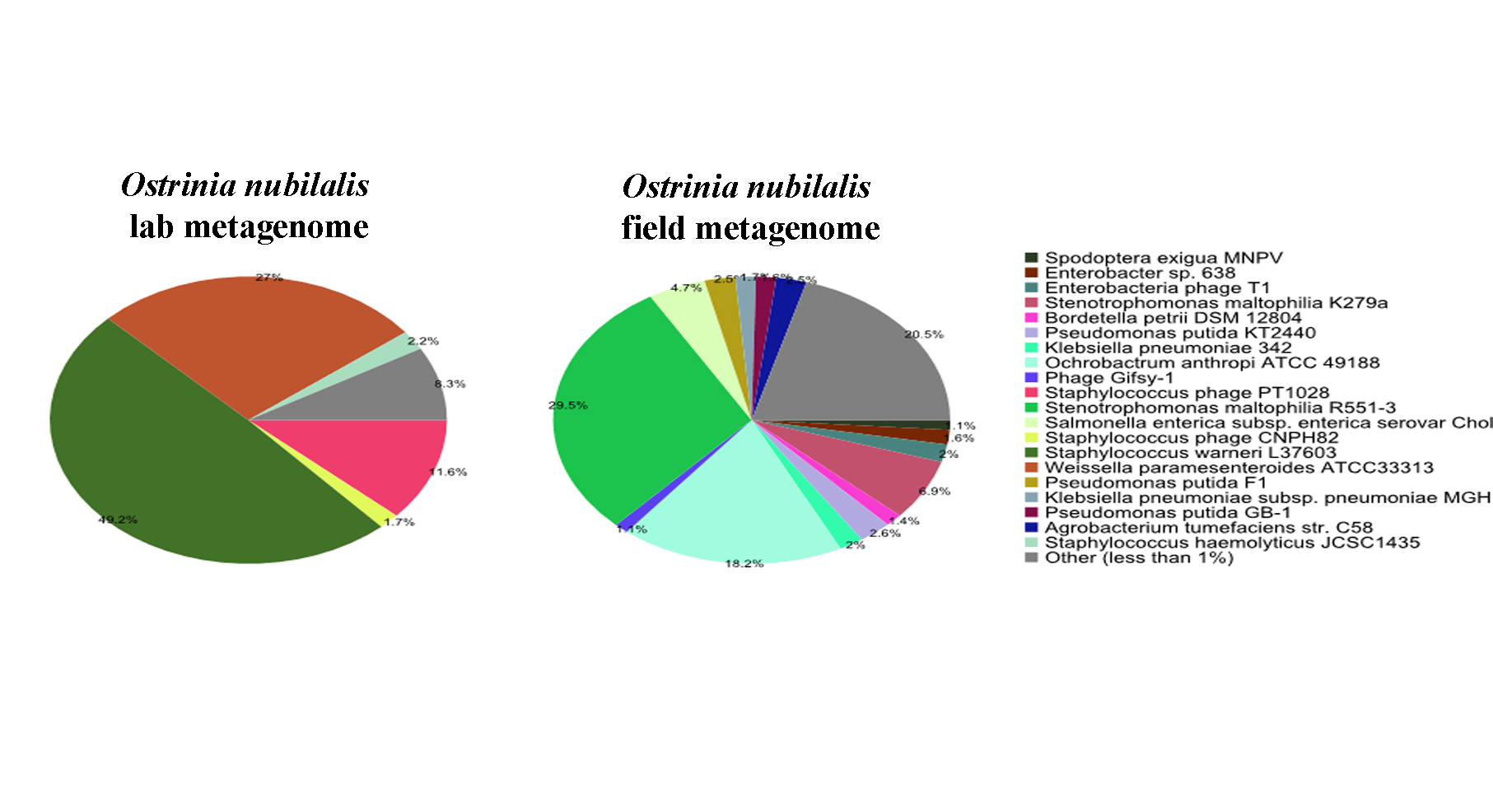

Supplement: Figure S3 — Genome relative abundance of lab and field O. nubilalis metagenomes based on the GAAS program results [34]. (TIF) [file pone.0021751.s003.tif]
